# Langerhans cell histiocytosis present in a 1-day-old girl: a case report

**DOI:** 10.1186/s13256-025-05485-8

**Published:** 2025-08-15

**Authors:** Yang Meng

**Affiliations:** Department of Pediatrics, The First People’s Hospital of Yancheng, Yancheng, Jiangsu Province China

**Keywords:** Langerhans-cell histiocytosis, Congenital rash, Infant, Newborn

## Abstract

**Background:**

Langerhans cell histiocytosis is a rare condition characterized by diverse clinical manifestations, ranging from cutaneous lesions to systemic involvement. Although Langerhans cell histiocytosis occurs infrequently in newborns, its diagnosis during this period presents significant challenges.

**Case presentation:**

A 1-day-old Chinese female infant presented with multiple red papules at birth and was diagnosed with Langerhans cell histiocytosis following a second skin biopsy. Despite symptomatic treatment, the child experienced recurrent fever and gastrointestinal bleeding. Tafinlar treatment was initiated at 2 months of age, which improved her symptoms.

**Conclusion:**

Prompt identification of Langerhans cell histiocytosis in newborns remains challenging owing to the disease’s complexity and varied clinical manifestations; thus, it is essential to advance pathological technologies for Langerhans cell histiocytosis detection.

## Introduction

Langerhans cell histiocytosis (LCH) encompasses a spectrum of idiopathic conditions characterized by the involvement of Langerhans cells. While its precise etiology remains unclear, it has been linked to neoplasia, immunological activation, and disorders of dendritic cells [1, 2]. Currently, LCH is recognized as a clonal proliferative disorder of Langerhans cells occurring within a hematopoietic cell microenvironment [2, 3]. This report presents a rare case of LCH in a full-term female neonate with multiple lesions.

## Case description

**Fig. 1:**
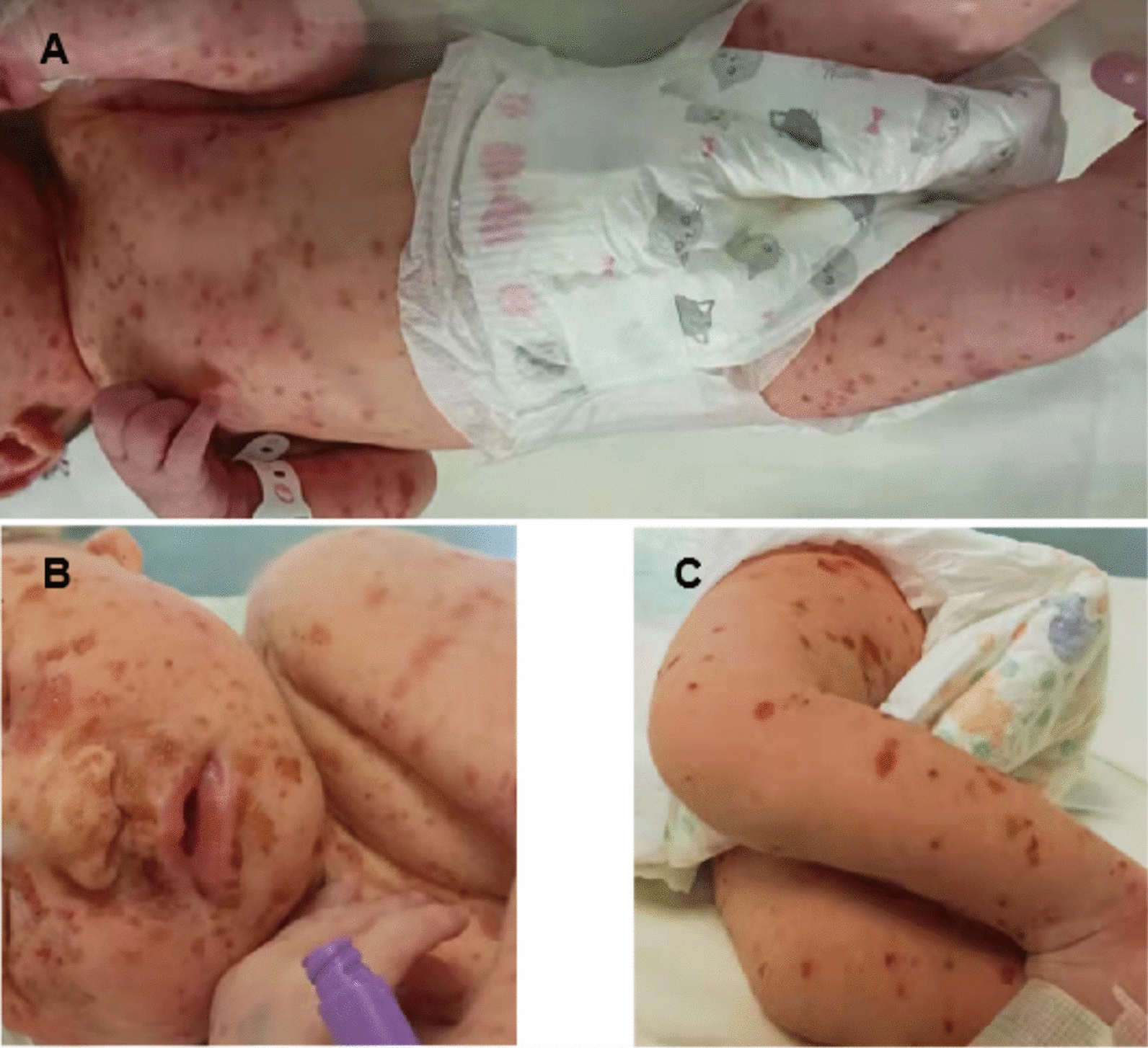
**A**–**C** Infant’s rash at birth with multiple erythematous cutaneous lesions. Lesions covered the entire body,except the palms and soles

A 1-day-old Chinese female infant presented with multiple red papules and cutaneous lesions with an ulcerated central area since birth (Fig. 1). She was delivered via cesarean section at 38 weeks of gestation due to her mother’s uterine scar. There was no significant family history, and her mother did not report using any systemic medications during pregnancy. The infant’s weight was 3.13 kg, and her Apgar scores at 1- and 5-minutes were 7 and 8, respectively. Upon admission to the neonatal intensive care unit on the second day, the infant’s vital signs were stable, and she was fed well. There were no signs of fever, weight loss, polyuria, or other systemic symptoms. Physical examination revealed multiple ill-defined lesions, measuring 5–10 mm in diameter, around her face, trunk, and extremities. In addition, a 0.5–1 cm node was observed inside the anus, which bled easily upon touch. Other physical findings, including the mucosal membranes, lymph nodes, liver, and spleen, appeared normal.

Initial differential diagnoses were syphilis, toxoplasma, rubella, cytomegalovirus, herpesvirus (TORCH) infections, Epstein–Barr virus (EBV), novel coronavirus, and human immunodeficiency virus (HIV). However, serological results for these infections were negative. Hepatic and renal function, blood electrolytes, chest radiograph, and blood cultures all reported normal findings, except for the laboratory values presented in Table 1. A skin biopsy from a lesion on the right lower limb revealed minor atypical hyperplasia of the squamous epithelium, along with karyokinesis and interstitial inflammation.

**Table 1:** All test results of the infant on day 3 after birth

Index		Outcome	Normal
PCT	ng/mL	18.95	0–0.5
WBC	10*9/L	3.50	5–14.5
Hb	10*12/L	126	138–218
RBC	10*12/L	3.94	5.2–6.4
PLT	10*9/L	57	150–300
HCT	%	38.3	45–65
Lymphocyte absolute value 10^9^/L		1.54	2.0–5.0
Neutrophil absolute value 10^9^/L		0.78	2.0–7.0
CD3 absolute count/uL		543	600–5000
CD4 absolute count/uL		318	400–3500

*PCT* procalcitonin; *WBC* white blood cell; *Hb* hemoglobin; *RBC* red blood cell; *PLT* platelet; *HCT* hematocrit

**Fig. 2:**
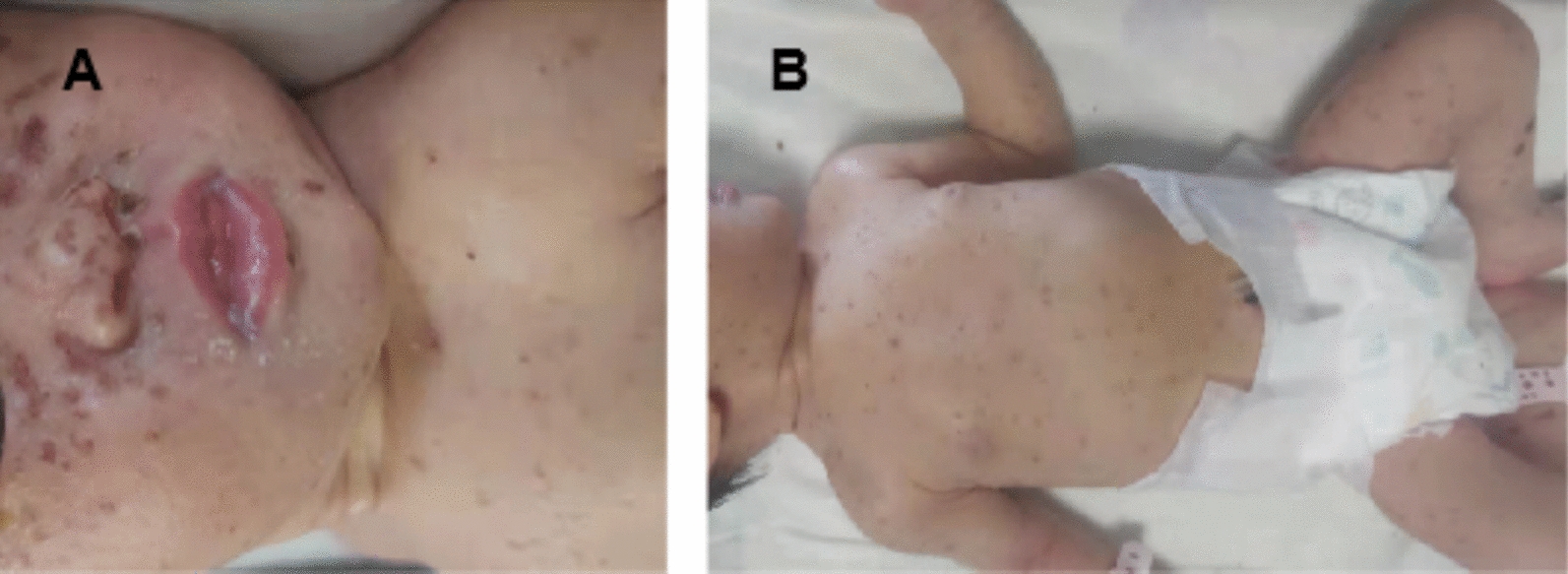
**A**, **B** Ulcerative lesion disappeared, replaced by a maculopapular and peeling scab

**Fig. 3:**
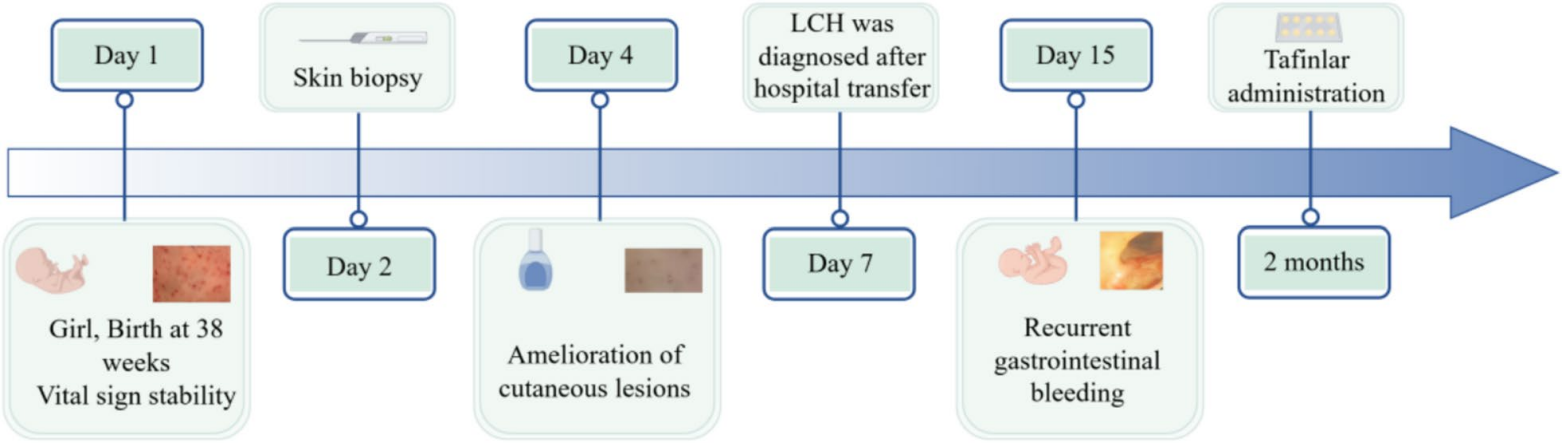
Timeline of clinical events and management

Upon consulting with a dermatologist, the infant was prescribed fusidic cream to prevent skin infection and human epidermal growth factor gel to promote healing. In addition, she received intravenous ceftazidime (50 mg/kg q12h), a broad-spectrum antibiotic commonly used for perinatal infections. Physical examination revealed the resolution of the ulcerative lesion, replaced by a maculopapular and peeling scab that persisted until the fourth day (Fig. 2). Following the transfer of the biopsy specimen to Shanghai Children’s Medical Center, LCH diagnosis was confirmed. Figure 3 illustrates the timeline of this case.

## Discussion

When a newborn presents with erythematous papulovesicular lesions, numerous differential diagnoses, including various infectious and neoplastic conditions, should be considered [1]. Similarly, infectious illnesses such as herpes simplex virus (HSV), varicella, TORCH infections, syphilis, congenital candidiasis, bullous impetigo, and listeriosis warrant consideration. Rare neoplastic conditions, including congenital leukemia, LCH, and neuroblastoma, may be overlooked in a newborn with erythematous cutaneous lesions [1].

In this case, diagnostic tests ruled out HIV, EBV, TORCH infections, syphilis, and septicemia. Although the procalcitonin (PCT) level was elevated, the infant demonstrated good feeding patterns, and the rash gradually improved with prescribed treatment, including medication and nursing interventions. Initial skin biopsy with hematoxylin and eosin (HE) staining and immunohistochemical staining did not yield a definitive diagnosis, as LCH was not initially considered owing to its infrequent occurrence in neonates. Characteristic manifestations of LCH include fever, rash, hepatomegaly, lymph node enlargement, and commonly associated progressive anemia, granulocytopenia, and thrombocytopenia [3, 4]. The elevated PCT level in this case suggested a possible prenatal infection, potentially compromising the infant’s cellular immune function. Furthermore, decreased levels of CD3 and CD4 indicated that LCH might have suppressed T-cell function, increasing susceptibility to infections. Additional research is necessary to elucidate the relationship between LCH and lymphocyte subsets. The suppression of all three blood components—white blood cells, red blood cells, and platelets—observed in laboratory tests corresponds with the invasive characteristics of LCH [3, 4]. The pathology department did not identify typical Langerhans cells on HE staining, and initial immunohistochemical staining failed to express key markers such as CD1a and S-100 [5]. Moreover, the presence of Birbeck granules, visible under electron microscopy, serves as a diagnostic criterion for LCH in pathology [5, 6]. Without comprehensive histology, immunocytochemistry, and electron microscopic examinations, LCH can be easily overlooked or misdiagnosed. This presents significant challenges for pediatric medical teams in establishing timely diagnoses and initiating appropriate treatment for neonates with LCH.

## Conclusion

Owing to progressive lesions affecting the digestive system mucosa, the patient required extended hospitalization (> 2 months) at Shanghai Children’s Medical Center. The severity of her condition necessitated continuous total parenteral nutrition throughout her hospital stay. During hematology department management, she developed recurrent fever and hematochezia, prompting comprehensive diagnostic evaluation including blood tests, genetic analysis, and bone marrow biopsy. Following Tafinlar administration, the patient showed significant clinical improvement. Her parents expressed gratitude for the multidisciplinary care and hope this case enhances pediatricians’ awareness of early LCH diagnosis. Ongoing monitoring through scheduled telephone consultations will assess her progress and ensure continuity of care.

## Data Availability

As the only resident physician, the pictures and experimental data provided were authentic.
